# Supplementary material for: Structural analysis and architectural principles of the bacterial amyloid curli
Source: Nat Commun. 2023 May 17;14:2822. doi: 10.1038/s41467-023-38204-2 (PMC10192328; doi:10.1038/s41467-023-38204-2)
Supplement: Supplementary file 1 — Supplementary Information [file 41467_2023_38204_MOESM1_ESM.pdf]

Supplementary Information to

Structural analysis and architectural principles of the bacterial amyloid curli

Author List Mike Sleutel[1,2*], Brajabandhu Pradhan[1,2], Alexander N. Volkov[1,3], Han Remaut[1,2*]

Affiliations

[1] Structural Biology Brussels, Vrije Universiteit Brussel, Pleinlaan 2, 1050 Brussels, Belgium

[2] Structural and Molecular Microbiology, VIB-VUB Center for Structural Biology, VIB, Pleinlaan 2, 1050 Brussels, Belgium

[3]Jean Jeener NMR Center, VIB, Pleinlaan 2, 1050 Brussel, Belgium.

[*]Correspondence to: Mike.Sleutel@vub.be, Han.Remaut@vub.be

Contents:

Supplementary Tables: 1-2

Supplementary Figures: 1-16

	#1 R15.5 (EMDB-NA) (PDB -NA)
**Data collection and processing**	
Magnification	60000
Voltage (kV)	300 kv
Electron exposure (e–/Å^2)	63.6
Defocus range (μm)	-0.5 to -3.5
Pixel size (Å)	0.766
Symmetry imposed	Helical (Twist= 180°, Rise = 72 Å)
Initial particle images (no.)	243391
Final particle images (no.)	64138
Map resolution (Å)	7.6
FSC threshold	0.143
**Model**	
Initial model used (PDB code)	Alphafold2
Model resolution (Å)	6.9
FSC threshold	0.143
Model composition	
Non-hydrogen atoms	7782
Protein residues	1065
B factors (Å^2)	
Protein	NULL
R.m.s. deviations	
Bond lengths (Å)	0.001
Bond angles (°)	0.480
Validation	
MolProbity score	1.09
Clashscore	3.04
Poor rotamers (%)	0.0
Ramachandran plot	
Favored (%)	100.0
Allowed (%)	0.0
Disallowed (%)	0.0
**Model vs Data**	
CC (mask)	0.43
CC (box)	0.58
CC (peaks)	0.34
CC (volume)	0.44

Supplementary Table 1: Cryo-EM data collection, refinement and validation statistics

A: Dali search of curlin R15.5 against PDB_25

PDB	Z-score	rmsd	lali	nres	%id	Description
**3p4g**	**18.1**	**9.0**	**257**	**313**	**12**	**MOLECULE: ANTIFREEZE PROTEIN**
**3pr7**	**16.1**	**2.2**	**201**	**292**	**6**	**MOLECULE: USPA1**
3suc	15.4	9.7	263	767	11	MOLECULE: PRENECK APPENDAGE PROTEIN
4xla	14.7	9.7	281	600	4	MOLECULE: TAIL SPIKE PROTEIN
5lw3	14.5	9.6	268	381	6	MOLECULE: POLY(BETA-D-MANNURONATE) C5 EPIMERASE 6
4mr0	14.4	9.7	263	456	6	MOLECULE: PLASMIN AND FIBRONECTIN-BINDING PROTEIN A
**5zru**	**14.1**	**9.9**	**276**	**576**	**8**	**MOLECULE: ALPHA-1,3-GLUCANASE**
6kqs	14.0	9.7	259	620	6	MOLECULE: LACTO-N-BIOSIDASE
2x3h	13.7	9.4	267	498	11	MOLECULE: K5 LYASE
1rmg	13.7	9.7	241	422	6	MOLECULE: RHAMNOGALACTURONASE A
5zku	13.7	9.6	258	443	8	MOLECULE: DFA-IIIASE
2odl	13.6	9.1	248	372	11	MOLECULE: ADHESIN
4rmx	13.5	10.1	261	602	8	MOLECULE: PHI92_GP150
2xc1	13.5	9.7	257	661	7	MOLECULE: BIFUNCTIONAL TAIL PROTEIN
4c2l	13.3	9.7	246	372	10	MOLECULE: ENDO-XYLOGALACTURONAN HYDROLASE A
6z7p	13.3	9.3	226	1025	9	MOLECULE: S-LAYER PROTEIN
7akv	13.0	9.4	257	421	8	MOLECULE: PLASMA PROTEASE C1 INHIBITOR
1dbg	12.7	9.5	267	481	6	MOLECULE: CHONDROITINASE B
5g5o	12.6	9.7	231	348	7	MOLECULE: LH3 HEXON-INTERLACING CAPSID PROTEIN
1hg8	12.6	9.6	238	349	9	MOLECULE: ENDOPOLYGALACTURONASE
7chu	12.5	9.6	238	517	9	MOLECULE: PUTATIVE PECTIN LYASE
6c72	12.3	9.6	256	579	8	MOLECULE: PARTICLE-ASSOCIATED GLYCOSIDE HYDROLASE
5ny0	12.2	9.4	216	343	11	MOLECULE: L. REUTERIS SRRP BINDING REGION
5awf	11.5	10.3	176	385	3	MOLECULE: FES CLUSTER ASSEMBLY PROTEIN SUFB
6tku	11.5	9.6	221	545	7	MOLECULE: DEPOLYMERASE KP32GP38
4nk6	11.4	9.6	249	424	9	MOLECULE: POLY(BETA-D-MANNURONATE) C5 EPIMERASE
7jw4	11.4	9.7	224	591	6	MOLECULE: GLYCOSIDE HYDROLASE FAMILY 110
5w5p	11.4	9.6	235	610	7	MOLECULE: TAIL FIBER PROTEIN
4dn7	11.3	9.6	193	406	2	MOLECULE: ABC TRANSPORTER, ATP-BINDING PROTEIN
5olq	11.2	9.7	224	427	5	MOLECULE: RHAMNOGALACTURONAN LYASE
3lmw	10.7	9.4	222	472	9	MOLECULE: IOTA-CARRAGEENASE, CGIA
5gqc	10.5	9.6	236	583	8	MOLECULE: LACTO-N-BIOSIDASE
6kfn	10.4	9.6	220	298	5	MOLECULE: ALGINATE LYASE
1rwr	10.3	8.9	186	297	9	MOLECULE: FILAMENTOUS HEMAGGLUTININ
3jqy	10.2	3.0	159	233	6	MOLECULE: POLYSIALIC ACID O-ACETYLTRANSFERASE
6bea	10.1	10.3	254	433	9	MOLECULE: AUTOTRANSPORTER PROTEIN UPAB
3jx8	10.0	9.1	181	247	12	MOLECULE: PUTATIVE LIPOPROTEIN
**3ult**	**9.3**	**1.6**	**111**	**114**	**9**	**MOLECULE: ICE RECRYSTALLIZATION INHIBITION PROTEIN-LIKE PRO**
6nzs	9.3	9.9	248	587	10	MOLECULE: DEXTRANASE
3wp8	8.9	7.2	162	317	7	MOLECULE: TRIMERIC AUTOTRANSPORTER ADHESIN
6fi2	8.9	9.4	184	375	7	MOLECULE: VEXL
4ay9	8.8	9.8	212	307	7	MOLECULE: GLYCOPROTEIN HORMONES, ALPHA POLYPEPTIDE
4lxr	8.8	11.1	244	755	7	MOLECULE: PROTEIN TOLL
6ody	8.7	10.1	272	693	5	MOLECULE: VACUOLATING CYTOTOXIN AUTOTRANSPORTER
3pet	8.7	8.9	161	218	3	MOLECULE: PUTATIVE ADHESIN
3vst	8.7	9.7	217	638	6	MOLECULE: XYLOSIDASE
4rt6	8.4	9.9	248	805	8	MOLECULE: HEME/HEMOPEXIN-BINDING PROTEIN
2f9c	8.3	3.6	172	320	13	MOLECULE: HYPOTHETICAL PROTEIN YDCK
2xqh	8.3	5.2	134	258	14	MOLECULE: IMMUNOGLOBULIN-BINDING PROTEIN EIBD
4pex	8.3	9.4	221	551	5	MOLECULE: PUTATIVE SECRETED PROTEIN
4fd0	8.2	9.7	240	391	3	MOLECULE: LEUCINE RICH HYPOTHETICAL PROTEIN

3g06	8.2	11.3	231	601	7	MOLECULE: SSPH2 (LEUCINE-RICH REPEAT PROTEIN)
1dab	8.0	9.5	253	539	10	MOLECULE: P.69 PERTACTIN
6tgf	7.9	11.0	227	687	5	MOLECULE: EXOPOLYSACCHARIDE BIOSYNTHESIS PROTEIN
1pxz	7.9	9.8	201	346	7	MOLECULE: MAJOR POLLEN ALLERGEN JUN A 1
6xja	7.9	9.2	203	1286	10	MOLECULE: IMMUNOGLOBULIN A1 PROTEASE
4oqt	7.8	12.0	239	475	5	MOLECULE: LEUCINE-RICH REPEAT AND IMMUNOGLOBULIN-LIKE DOMAI
4fs7	7.7	9.9	230	383	4	MOLECULE: UNCHARACTERIZED PROTEIN
3oja	7.7	11.0	235	534	3	MOLECULE: LEUCINE-RICH IMMUNE MOLECULE 1
3d8v	7.6	8.5	163	477	4	MOLECULE: BIFUNCTIONAL PROTEIN GLMU
4kh3	7.6	9.6	224	492	8	MOLECULE: ANTIGEN 43
3bz5	7.5	11.5	240	450	6	MOLECULE: INTERNALIN-J
6nyf	7.4	9.7	233	750	6	MOLECULE: VACUOLATING CYTOTOXIN AUTOTRANSPORTER
4peu	7.4	9.3	177	250	4	MOLECULE: UNCHARACTERIZED PROTEIN
5gkd	7.4	9.7	251	726	6	MOLECULE: ALYGC
4dt5	7.2	3.7	107	143	12	MOLECULE: ANTIFREEZE PROTEIN
2zu0	7.1	9.9	182	416	6	MOLECULE: PROTEIN SUFD
3bh6	7.1	12.1	147	314	5	MOLECULE: ADP-RIBOSYLATION FACTOR-LIKE PROTEIN 3
5t5i	7.0	9.5	173	269	13	MOLECULE: TUNGSTEN FORMYLMETHANOFURAN DEHYDROGENASE SUBUNIT
1qjv	6.9	9.5	177	342	7	MOLECULE: PECTIN METHYLESTERASE
3grh	6.5	9.5	183	397	4	MOLECULE: ACYL-COA THIOESTER HYDROLASE YBGC
3ak5	6.5	10.4	212	970	6	MOLECULE: HEMOGLOBIN-BINDING PROTEASE HBP
4mxn	6.4	9.4	143	226	8	MOLECULE: UNCHARACTERIZED PROTEIN
6n2c	6.3	9.3	182	409	9	MOLECULE: TAPIRIN
6nyr	6.3	11.4	247	681	10	MOLECULE: CROV588
4fcg	6.3	9.2	196	296	6	MOLECULE: UNCHARACTERIZED PROTEIN
1wwl	6.0	9.7	173	312	3	MOLECULE: MONOCYTE DIFFERENTIATION ANTIGEN CD14

B: Dali search of curlin R15.5 against human AF2 proteome

ID	Z-score	rmsd	lali	nres	%id	Description
**e3jb**	**34.2**	**8.2**	**352**	**1400**	**7**	**HUMAN:AF-Q685J3-F3 MUCIN-17**
**e5gb**	**28.4**	**8.3**	**312**	**1400**	**9**	**HUMAN:AF-Q7Z5P9-F22 MUCIN-19**
e4d7	27.4	7.4	310	1410	14	HUMAN:AF-Q9P2E9-F1 RIBOSOME-BINDING PROTEIN 1
**e4r1**	**23.7**	**8.8**	**272**	**590**	**6**	**HUMAN:AF-Q6UWP8-F1 SUPRABASIN**
**e87d**	**22.7**	**9.7**	**268**	**452**	**6**	**HUMAN:AF-Q8N7P7-F1 UNCHARACTERIZED PROTEIN FLJ40521**
fcus	21.2	8.7	300	558	10	HUMAN:AF-Q13117-F1 DELETED IN AZOOSPERMIA PROTEIN 2
e9rk	19.5	8.3	237	626	14	HUMAN:AF-A0A140T8X8-F1 MUCIN-21
e8j0	17.8	10.0	287	1400	5	HUMAN:AF-Q02817-F10 MUCIN-2
fgb6	17.1	8.5	185	251	8	HUMAN:AF-E2RYF7-F1 PROTEIN PBMUCL2
e3xd	16.0	8.3	167	195	2	HUMAN:AF-Q9BYR4-F1 KERATIN-ASSOCIATED PROTEIN 4-3
**e6zy**	**15.8**	**8.7**	**144**	**146**	**4**	**HUMAN:AF-P59991-F1 KERATIN-ASSOCIATED PROTEIN 12-2**
e99f	15.3	8.4	220	1052	5	HUMAN:AF-A0A0U1RQI7-F1 KRUPPEL-LIKE FACTOR 18
e356	14.8	9.3	278	564	13	HUMAN:AF-Q9UF83-F1 UNCHARACTERIZED PROTEIN DKFZP434B061
fgvk	14.4	9.7	188	2530	8	HUMAN:AF-P16112-F1 AGGRECAN CORE PROTEIN
fboo	14.2	9.2	216	392	5	HUMAN:AF-Q8N0U4-F1 PROTEIN FAM185A
e77y	13.8	9.6	292	1400	5	HUMAN:AF-Q09666-F8 NEUROBLAST DIFFERENTIATION-ASSOCIATED PROTEIN AHN
ffsm	13.8	10.3	304	904	4	HUMAN:AF-Q9NXZ1-F1 SARCOMA ANTIGEN 1
fdpk	13.5	9.8	278	1431	5	HUMAN:AF-Q12816-F1 TROPHININ
e2r0	13.3	8.8	200	2290	3	HUMAN:AF-Q5H9R4-F1 ARMADILLO REPEAT-CONTAINING X-LINKED PROTEIN 4
e0ms	12.6	9.2	208	1400	4	HUMAN:AF-Q02505-F8 MUCIN-3A
e1kv	12.3	9.5	264	1400	7	HUMAN:AF-Q86WI1-F12 FIBROCYSTIN-L
fdt2	11.9	9.7	187	1400	9	HUMAN:AF-Q8IVF2-F15 PROTEIN AHNAK2
ffv9	11.3	9.5	270	2061	9	HUMAN:AF-A0A494C071-F1 UNCHARACTERIZED PROTEIN
fde4	9.6	9.8	287	1361	5	HUMAN:AF-Q8WUJ3-F1 CELL MIGRATION-INDUCING AND HYALURONAN-BINDING PR
e0ru	9.6	9.3	263	1400	8	HUMAN:AF-P08F94-F7 FIBROCYSTIN
e401	9.5	13.0	157	704	4	HUMAN:AF-Q96NY7-F1 CHLORIDE INTRACELLULAR CHANNEL PROTEIN 6
e6dz	9.1	10.5	206	956	3	HUMAN:AF-Q9UK96-F1 F-BOX ONLY PROTEIN 10

e66q	9.0	8.7	257	1357	13	HUMAN:AF-Q96Q06-F1 PERILIPIN-4
e8oe	9.0	9.6	205	1400	5	HUMAN:AF-Q02505-F5 MUCIN-3A
ezu8	9.0	8.6	196	744	9	HUMAN:AF-Q9NQZ3-F1 DELETED IN AZOOSPERMIA PROTEIN 1
ffc4	8.8	9.5	196	446	6	HUMAN:AF-Q8N1N5-F1 PUTATIVE PROTEIN CRIPAK
fa4m	8.7	9.3	271	1400	9	HUMAN:AF-Q09666-F18 NEUROBLAST DIFFERENTIATION-ASSOCIATED PROTEIN AHN
fe2b	8.4	9.4	206	784	7	HUMAN:AF-Q6XPR3-F1 REPETIN
e7bx	8.4	9.7	299	1383	6	HUMAN:AF-Q9UHN6-F1 CELL SURFACE HYALURONIDASE
e6eg	8.3	10.6	219	764	7	HUMAN:AF-P16473-F1 THYROTROPIN RECEPTOR
fc7j	8.2	12.6	237	620	7	HUMAN:AF-Q96FE5-F1 LEUCINE-RICH REPEAT AND IMMUNOGLOBULIN-LIKE DOMAI
e4st	7.7	10.9	249	907	5	HUMAN:AF-O75473-F1 LEUCINE-RICH REPEAT-CONTAINING G-PROTEIN COUPLED
ffpw	7.3	10.8	195	699	5	HUMAN:AF-P22888-F1 LUTROPIN-CHORIOGONADOTROPIC HORMONE RECEPTOR
e95j	7.2	8.8	160	288	4	HUMAN:AF-Q6L8H1-F1 KERATIN-ASSOCIATED PROTEIN 5-4
e39t	7.1	9.4	214	1255	6	HUMAN:AF-P15941-F1 MUCIN-1
e1u0	7.1	10.2	245	1035	4	HUMAN:AF-Q5HY64-F1 PUTATIVE PROTEIN FAM47C
e30k	7.1	9.7	159	517	5	HUMAN:AF-A8MUX0-F1 KERATIN-ASSOCIATED PROTEIN 16-1
e3ff	6.9	10.4	217	649	7	HUMAN:AF-Q9NZU0-F1 LEUCINE-RICH REPEAT TRANSMEMBRANE PROTEIN FLRT3
feno	6.8	10.9	185	695	7	HUMAN:AF-P23945-F1 FOLLICLE-STIMULATING HORMONE RECEPTOR
ffvd	6.7	9.0	180	927	4	HUMAN:AF-Q86XK2-F1 F-BOX ONLY PROTEIN 11
fdiq	6.6	11.5	164	616	3	HUMAN:AF-A6NJ88-F1 PUTATIVE SAGE1-LIKE PROTEIN
e6q6	6.4	9.6	157	1385	5	HUMAN:AF-Q96M83-F1 COILED-COIL DOMAIN-CONTAINING PROTEIN 7
e9t1	6.3	10.8	223	716	4	HUMAN:AF-Q6UXK5-F1 LEUCINE-RICH REPEAT NEURONAL PROTEIN 1
feji	6.3	8.4	186	1819	2	HUMAN:AF-Q96RT7-F1 GAMMA-TUBULIN COMPLEX COMPONENT 6
ez5c	6.2	11.0	203	421	4	HUMAN:AF-Q99983-F1 OSTEOMODULIN
e7ak	6.0	10.2	200	380	6	HUMAN:AF-Q9BXN1-F1 ASPORIN

Supplementary Table 2: Structural analogs of the curlin β-solenoid fold. A structural similarity search of the PDB_25 (A) and the human Alphafold protein structure database (B) was performed using the Dali server (ref) and the model of the CS-class CsgA representative A0A0E3UX01 (i.e. R15.5). For clarity, the tabulated results are rarefied to remove redundant or closely related entries. Representative proteins highlighted in bold, are shown in Supplementary Fig. 15.

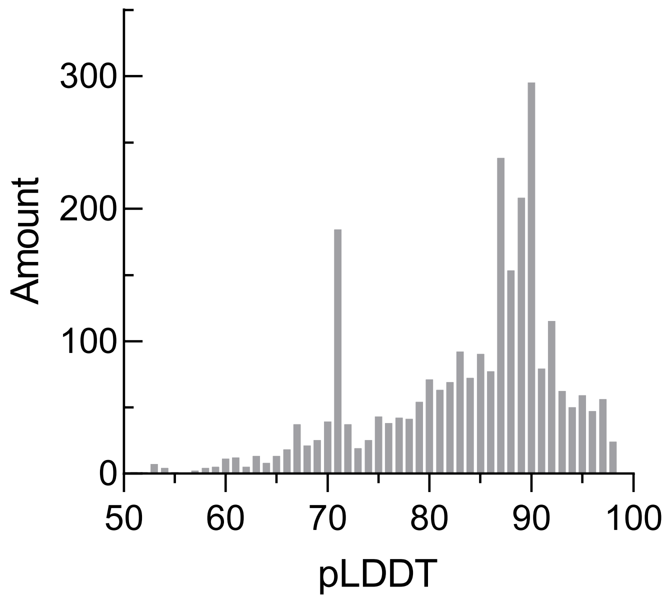

Supplementary Figure 1: Histogram of the reported pLDDT values for the total dataset of predicted CsgA structures. DeepMind reports pLDDT > 90 as high accuracy predictions, between 70 and 90 as good backbone predictions, and pLDDT < 70 as low confidence and to be treated with caution. Source data are provided as a Source Data file.

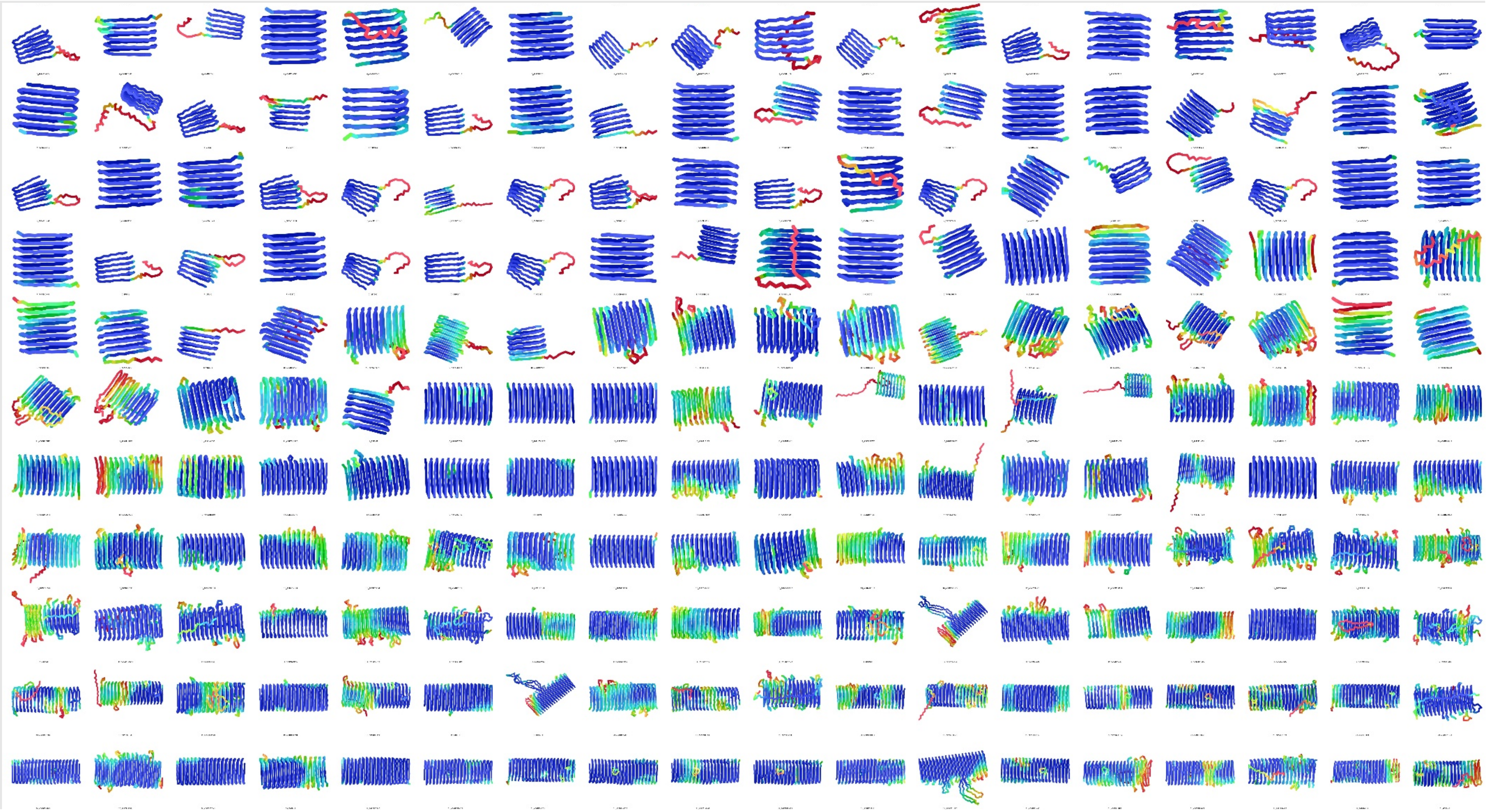

Supplementary Figure 2: Representative collage of a subset of CsgA homologue models produced by AlphaFold2, covering the structural diversity in the database. Colour coding according to pLDDT values.

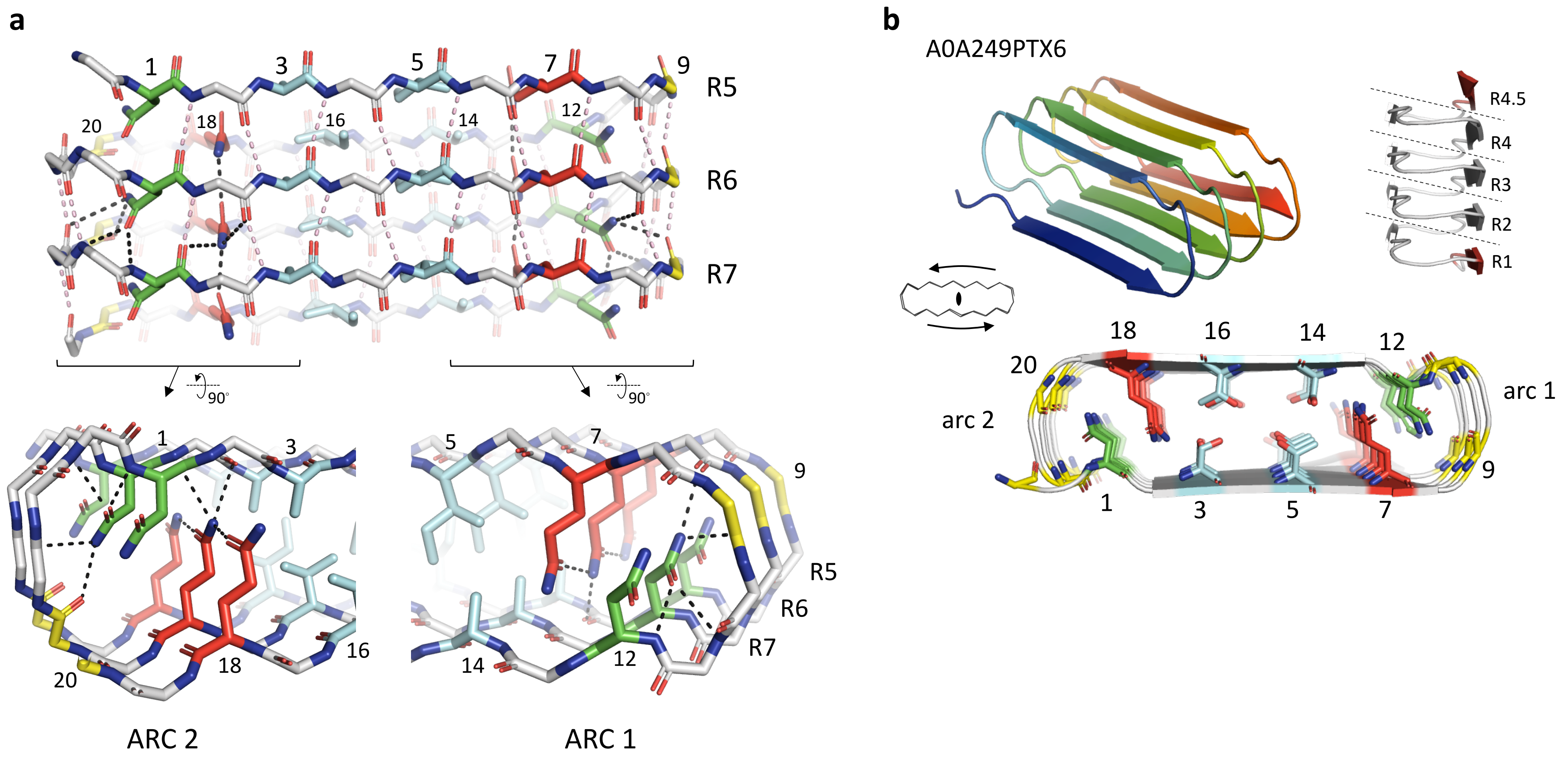

Supplementary Figure 3: Structural analysis of curlin repeat contacts. **(a)** Stick representation of three representative curlin repeats (R5-R6-R7) of *Pontibacter korlensis* CsgA (i.e. R15.5) shown in frontal view (top, with motif a facing forward), or in a close-up axial view of β-arc 1 (lower right) and β-arc 2 (lower left). For clarity, sidechains of surface exposed residues are not shown. N, Q and Ψ in motif a and B are colored green, red and sky blue, resp. Gly are colored yellow. Main chain H-bonds between consecutive curlin repeats are shown as pink dash. H-bonds formed by N, and Q in the curlin repeats are colored black (shown for R6 only). **(b)** Predicted structure of CsgA-like protein A0A249PTX6 of *Sinorhizobium fredii* shown in ribbon representation (upper left, blue to red from N- to C-term), in axial view (bottom), with inward facing residues shown in stick representation and colored as in (a), or in side view (upper right), with dashed lines indicating consecutive curlin repeats (labeled R1 to R4, terminating in half-repeat R4.5).

R15.5 RIGHT-HANDED

R15.5 LEFT-HANDED

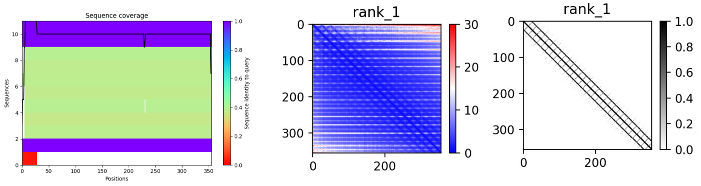

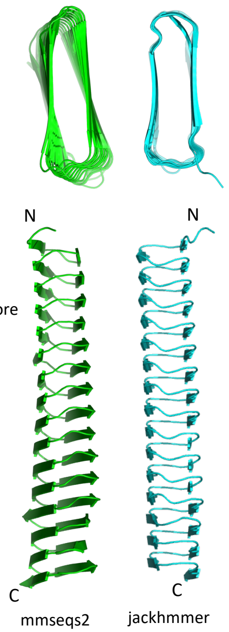

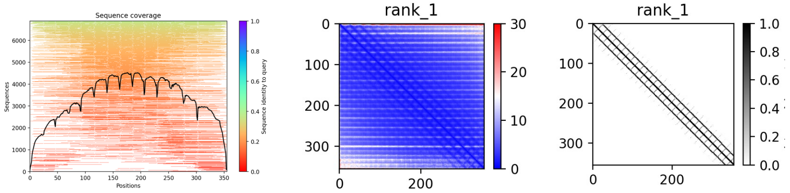

pLDDT 92.84

pTMscore 0.9118

pLDDT 93.68

pTMscore: 0.8846

All-Atom	Clashscore, all atoms:	0.41		99th percentile (N=1784, all resolutions)
Contacts	Clashscore is the number of serious steric overlaps (> 0.4 Å) per 1000 atoms.			
Protein	Poor rotamers	3	1.10%	Goal: <0.3%
Geometry	Favored rotamers	265	97.07%	Goal: >98%
	Ramachandran outliers	2	0.57%	Goal: <0.05%
	Ramachandran favored	347	98.30%	Goal: >98%
	Rama distribution Z-score	0.04 ± 0.44		Goal: abs(Z score) < 2
	MolProbity score	0.68		100th percentile (N=27675, 0Å - 99Å)
	Cβ deviations >0.25Å	0	0.00%	Goal: 0
	Bad bonds:	0 / 2616	0.00%	Goal: 0%
	Bad angles:	40 / 3562	1.12%	Goal: <0.1%
Peptide Omegas	Cis Prolines:	0 / 0	0.00%	Expected: ≤1 per chain, or ≤5%
	Twisted Peptides:	3 / 354	0.85%	Goal: 0
Low-resolution Criteria	CaBLAM outliers	24	6.80%	Goal: <1.0%
	CA Geometry outliers	2	0.57%	Goal: <0.5%

All-Atom	Clashscore, all atoms:	0.41		99th percentile (N=1784, all resolutions)
Contacts	Clashscore is the number of serious steric overlaps (> 0.4 Å) per 1000 atoms.			
Protein	Poor rotamers	1	0.37%	Goal: <0.3%
Geometry	Favored rotamers	266	97.44%	Goal: >98%
	Ramachandran outliers	0	0.00%	Goal: <0.05%
	Ramachandran favored	347	98.30%	Goal: >98%
	Rama distribution Z-score	-1.21 ± 0.42		Goal: abs(Z score) < 2
	MolProbity score	0.65		100th percentile (N=27675, 0Å - 99Å)
	Cβ deviations >0.25Å	0	0.00%	Goal: 0
	Bad bonds:	0 / 2616	0.00%	Goal: 0%
	Bad angles:	39 / 3562	1.09%	Goal: <0.1%
Peptide Omegas	Cis Prolines:	0 / 0	0.00%	Expected: ≤1 per chain, or ≤5%
Low-resolution Criteria	CaBLAM outliers	28	8.00%	Goal: <1.0%
	CA Geometry outliers	21	5.98%	Goal: <0.5%

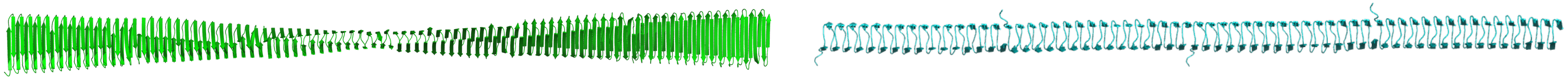

Supplementary Figure 4: Structural analysis of AMBER relaxed, right- and left-handed AF2 models of R15.5. Top panels as produced by ColabFold (https://colab.research.google.com/github/sokrypton/ColabFold/): MSA coverage (left); predicted alignment error (middle); Predicted contacts (right). Tables: MolProbity report for each model taken from http://molprobity.biochem.duke.edu/. The AF2 pLDDT and pTMscores for the right- and left-handed model are nearly identical, as well as the contact map and alignment error matrix. Molprobity scores for both structures are excellent, i.e. 0.68 and 0.65 (100th percentile of the reference database) and nearly identical. The only meaningful difference between both models -apart from the chirality of the fold- is the twist of the of the β-solenoid. The left-handed R15.5 has no measurable twist, whereas the right-handed model has a 17° twist from N- to C-terminus. Simulated fibril models based on right- or left-handed protomers are shown below.

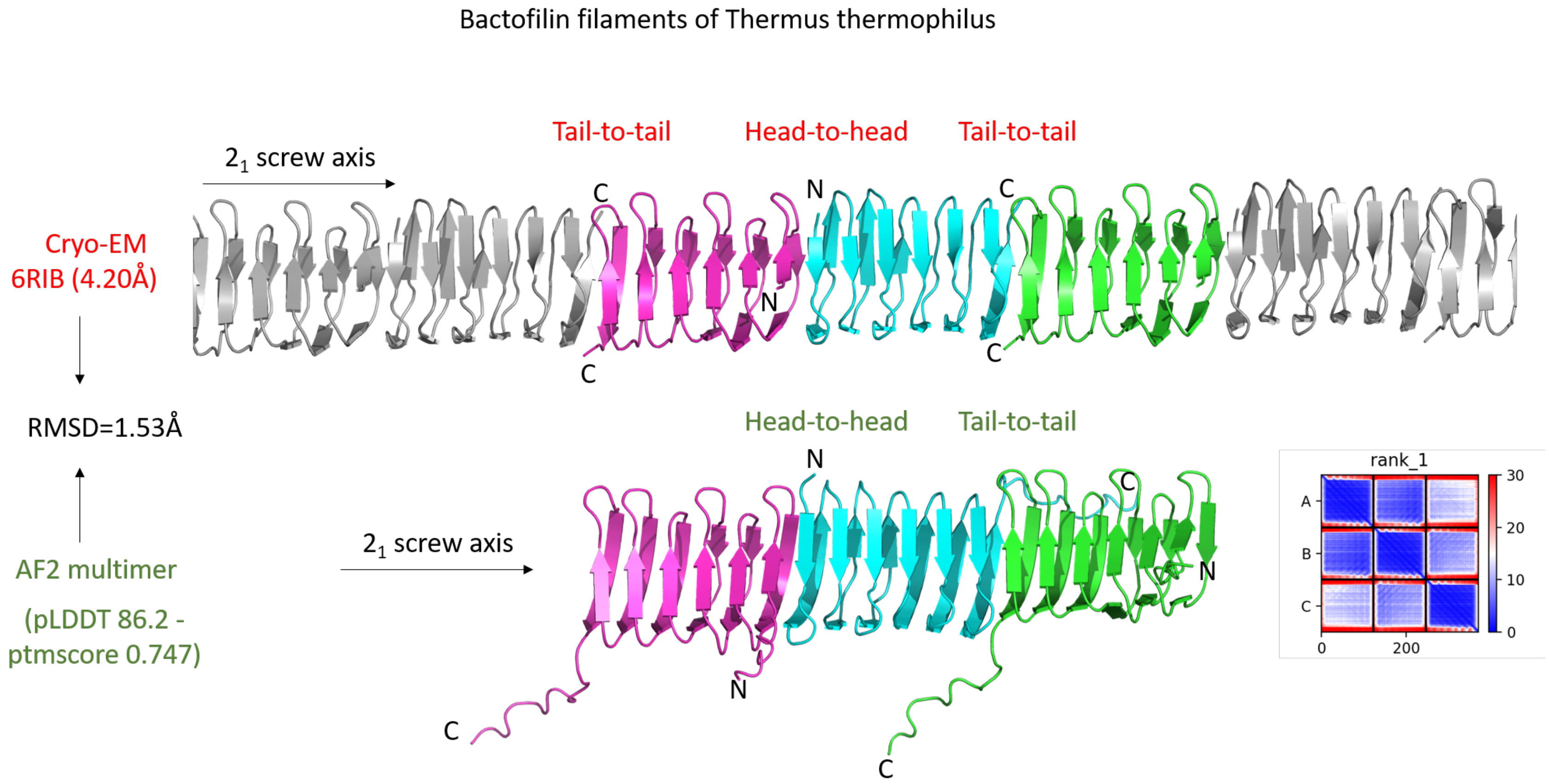

Supplementary Figure 5: Benchmarking example of the proposed AF2 methodology to predict filament structures: we compare an experimental cryo-EM structure (Bactofilin filaments of *Thermus thermophilus*) with a trimer that was predicted using AF2 multimer. Overall, there is excellent agreement between the predicted and experimental structure, with an RMSD of 1.53Å. AF2 accurately predicts the apolar nature of the filaments (i.e. succession of head-to-head and tail-to-tail interfaces) as well as the helical nature (i.e. screw axis). Note that the cryoEM structure was deposited on 2019-04-23, whereas AF2 is trained on protein chains in the PDB released before 2018-04-30.

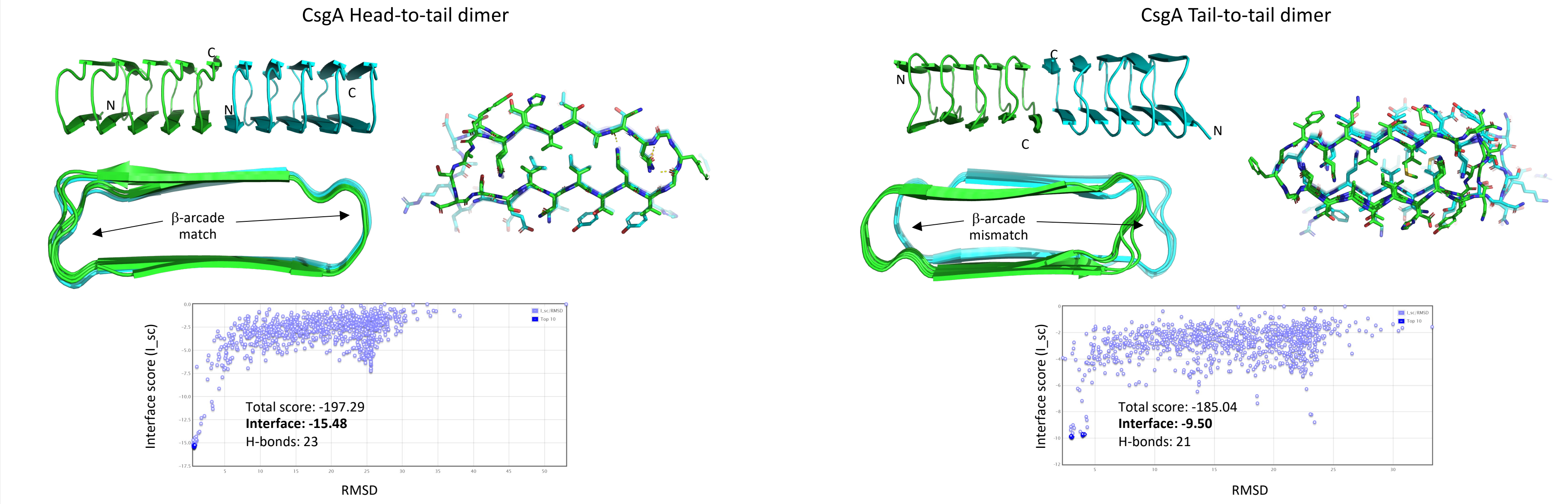

Supplementary Figure 6: *In silico* models of a head-to-tail and tail-to-tail CsgA dimer. The tail-to-tail dimer model was generated by initial manual placement of two CsgA AF2 monomers (without N_{22}) in a 'tail-to-tail' configuration, followed by local docking using RosettaDock. For the head-to-tail dimer we used the AF2 dimer model as an input structure for RosettaDock. Both models shown here correspond to their respective top-ranking structures that were generated by the ROSIE server. We plot the interface score (I_sc) for 1000 randomly placed models and show the total score, I_sc value and number of putative H-bonds for the top ranking models, respectively. The head-to-tail dimer packs in a parallel fashion resulting in β-sheet augmentation, and uninterrupted matching of the β-arcades, whereas the tail-to-tail dimer packs anti-parallel, triggering a lateral offset of 1 residue between both strands at the interface, and poor contacts at the arcades as a result. Putative H-bonds were detected using ChimeraX v1.2 using relaxed settings.

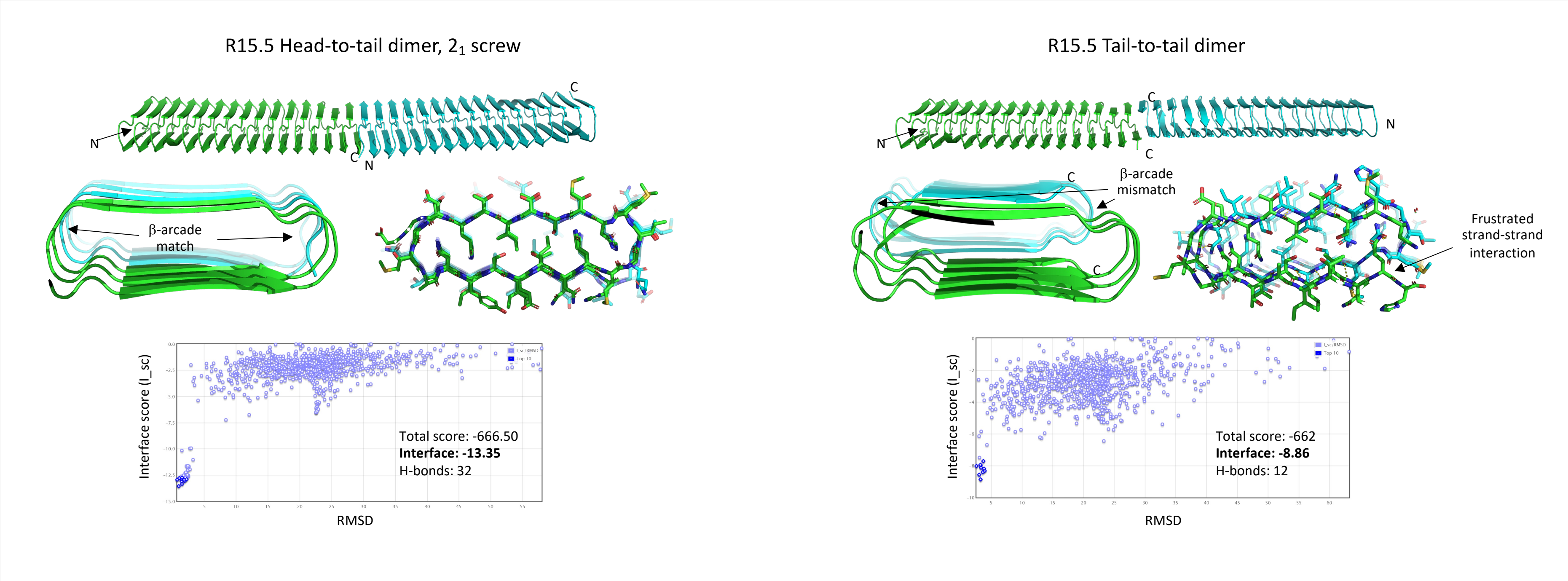

Supplementary Figure 7: *In silico* models of a head-to-tail and tail-to-tail R15.5 dimer. The tail-to-tail dimer model was generated by initial manual placement of two R15.5 AF2 monomers in a 'tail-to-tail' configuration, followed by local docking using RosettaDock. For the head-to-tail dimer we used the AF2 dimer model as an input structure for RosettaDock. Both models shown here correspond to their respective top-ranking structures that were generated by the ROSIE server. We plot the interface score (I_sc) for 1000 randomly placed models and show the total score, I_sc value and number of putative H-bonds for the top ranking models, respectively. The head-to-tail dimer packs in a parallel fashion resulting in β-sheet augmentation, and uninterrupted matching of the β-arcades. Similarly to the tail-to-tail dimer for CsgA, the tail-to-tail R15.5 dimer packs anti-parallel, triggering a lateral offset of 1 residue between both strands at the interface, and poor contacts at the arcades as a result. Putative H-bonds were detected using ChimeraX v1.2 using relaxed settings.

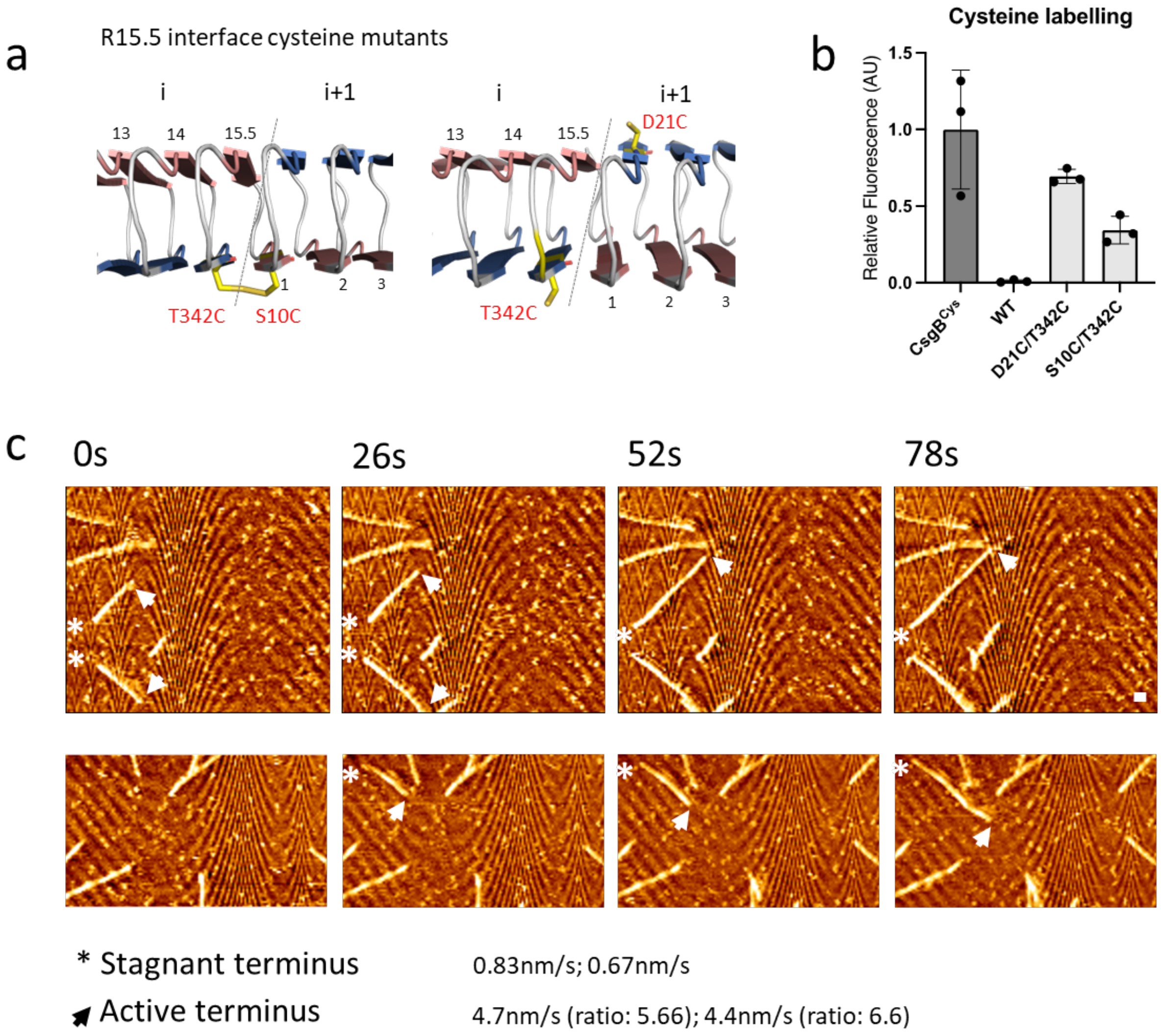

Supplementary Figure 8: Disulfide crosslinking across R15.5 subunit interface. (a)(b) Free thiol labelling in R15.5 double Cys mutants with a Cys in motif a (S10C) or motif b (T24C) at the N-terminus of subunit i+1 and in motif b (T342C) at the penultimate repeat of subunit i. Data show relative fluorescent signal at 680 nm of 100 ng WT R15.5, and its double cys mutants D21C/T342C and S10C/T342C, with free thiols labeled with IRDye 680 maleimide, and normalized to the fluorescence signal of IRDye 680 labelled $CsgB^{Cys}$, as non-oxidizing control. n=3 biological replicates. Data are presented as mean +/- SD. The low residual free thiol labelling in S10C/T342C indicates Cys are involved in the formation of motif a – b disulfides, in accordance with the predicted head-to-tail packing with 2_1 screw axis (panel a, left). A reduction in free thiol labelling in D21C/T342C may result from non-specific cysteine oxidation in solution, and/or translational head-to-tail packing of monomers as a result of the expulsion of the first (i.e. R1 motif a, labeled 1) or last (i.e. R15.5 motif b, labeled 15.5) strand from the monomer β-solenoid. Source data are provided as a Source Data file. **(c)** Time-lapse AFM imaging of R15.5 fibrils formed *in situ*: R15.5 protofibrils exhibit polar extension of their termini. For this we measured the extension rate at both poles of single fibrils and identify a fast and slow growing terminus. Following the two marked fibers in panel (c) we obtain growth rates of 4.7nm/s and 4.4nm/s for the fast growing pole, and 0.83nm/s and 0.67nm/s for the slow growing pole. Scale bar in panel c at 78s is 100nm.

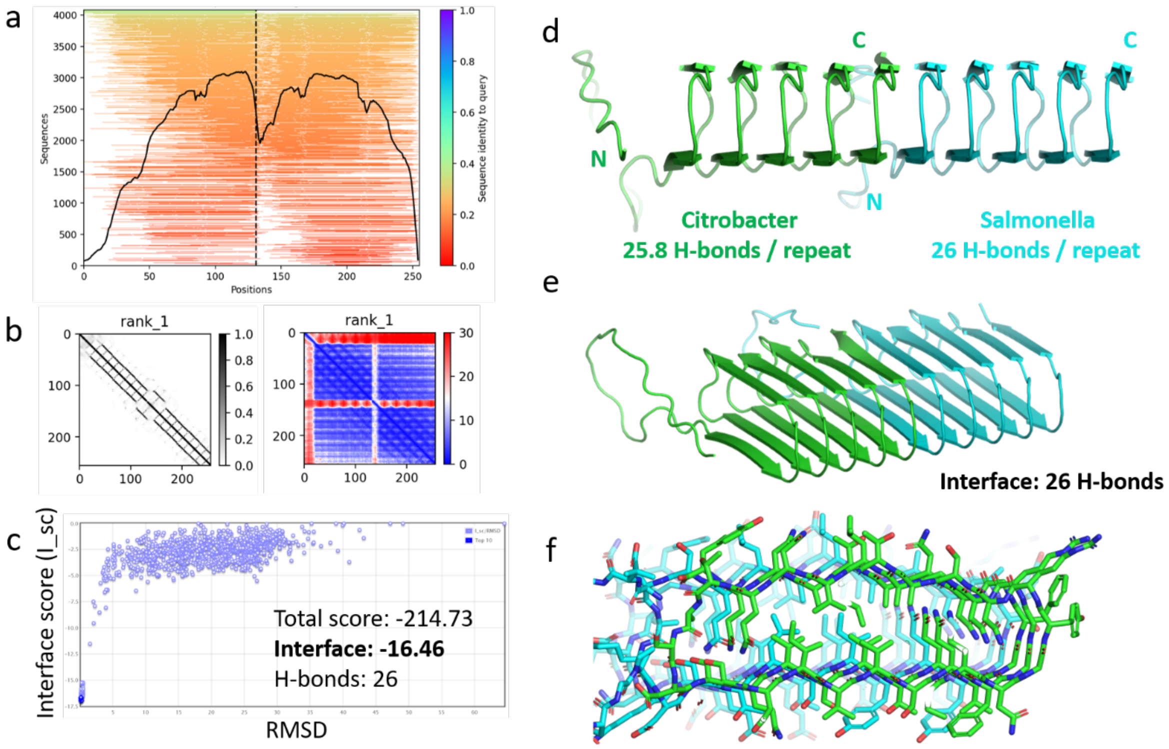

Supplementary Figure 9: Predicted heteromeric dimer of CsgA monomers from *Citrobacter* and *Salmonella*. **(a)** Multiple sequence alignment; **(b)** Predicted contacts and alignment error; **(c)** Rosettadock interface score as a function of the RMSD with respect to the AF2 input structure; **(d)** Side-view and **(e)** tilt-view of a head-to-tail heteromeric dimer of CsgA-CsgA *Citrobacter*-*Salmonella* as predicted by AF2. Each respective CsgA monomer has 25.8 and 26 predicted H-bonds per repeat on average. The interface between both subunits entails 26 putative H-bonds; **(f)** On-axis view in stick representation highlighting the uninterrupted steric zipper columns across the dimer interface.

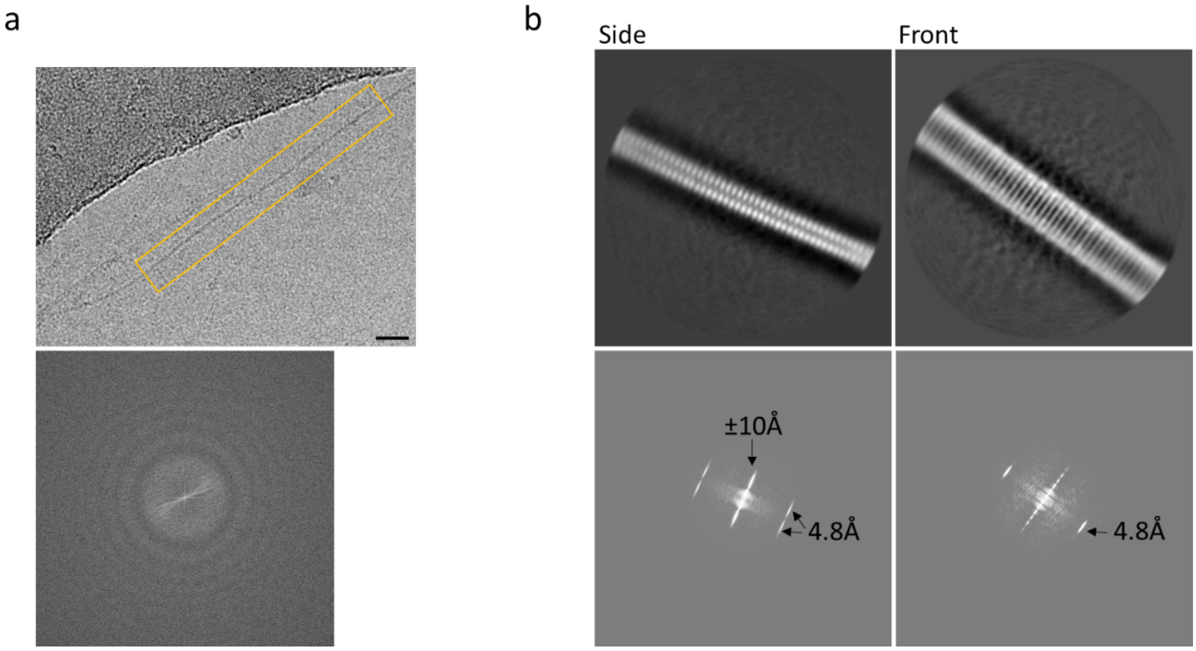

Supplementary Figure 10: (a) Boxed (x by y pixels at 0.784 Å/pix) single R15.5 fiber from an unprocessed cryoEM micrograph (upper), and corresponding power spectrum (lower) showing a lack of discernable features such as a helical or monomer periodicity at single fiber level. Scale-bar is 20nm **(b)** Side and front view 2D class averages (box-size:230 Å x 230 Å) and corresponding power spectra output by RELION 3.1.3 at a sigma contrast value of 5.0. Individual cryoEM images and 2D classes are representative for at least 5 independent sample preparations.

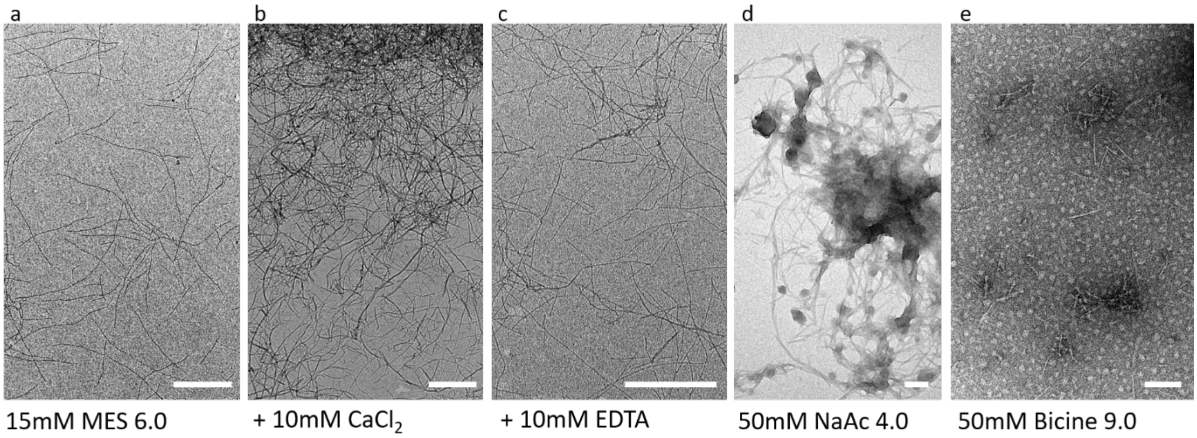

Supplementary Fig. 11: Negative stain TEM images of R15.5 fibrils formed in **(a)** 15mM MES 6.0; **(b)** 15mM MES pH 6.0 with 10mM $CaCl_2$; **(c)** 15mM MES 6.0 with 10mM EDTA; **(d)** 50mM NaAc pH 4.0; **(e)** 50mM Bicine pH 9.0. R15.5 monomer (5μM) stocks in 8M urea were desalted using a zeba spin column to the corresponding buffer and incubated at RT ON. The net charge of a folded R15.5 monomer was calculated using PROPKA (https://server.poissonboltzmann.org/) to be -12.35, -55.21 and -72.82 at pH 4.0, 6.0 and 9.0. The presence of 10mM $CaCl_2$ leads to enhanced aggregation of protofibrils into larger bundles. The 10mM EDTA sample was prepared to test if chelation of trace amounts of divalent cations or surface bound cations that were co-purified with R15.5 from the cellular lysate could lead to inhibition of curlin formation. The 50mM NaAc pH 4.0 sample reveals a mixed curlin / aggregate population, suggesting that a reduction of the surface charge facilitates non-amyloid aggregation of R15.5. Conversely, the increased net negative charge at pH 9.0 does not inhibit curlin formation. All cryoEM images are representative of at least 3 independent sample preparations.

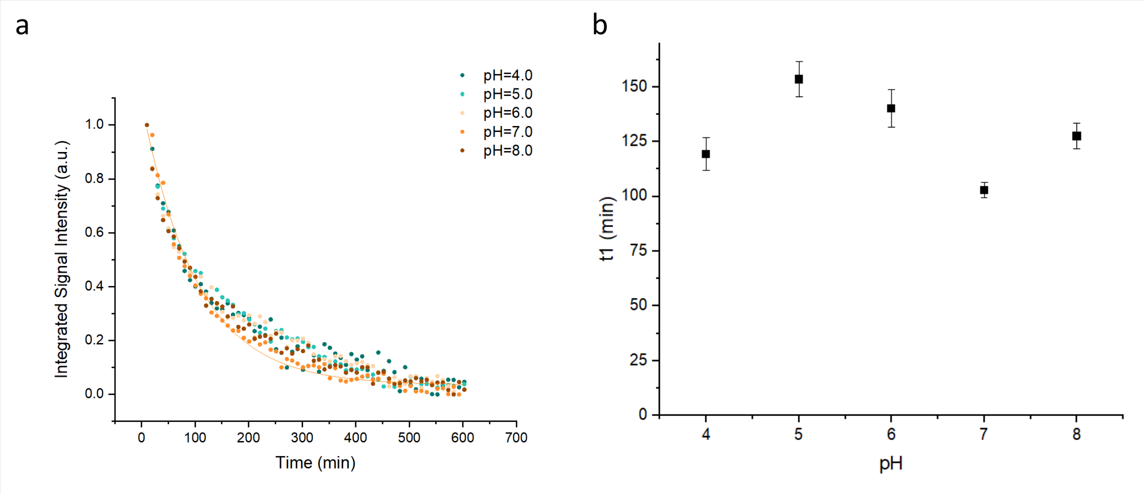

Supplementary Figure 12. R15.5 polymerization followed by 1D ^{1}H NMR. (a) Integrated 1D ^{1}H NMR signal intensity of 100 µM unfolded R15.5 monomers followed over time after fresh desalting from 50mM potassium phosphate, 250 mM imidazole, 8M urea, pH 7.5 to McIlvaine buffer (citrate-phosphate buffer) at different pH (4.0 to 8.0). 1D ^{1}H spectra were integrated over the 0.6 - 0.9 ppm spectral region and normalized with respect to t=0. ^{1}H proton signals decay as the unfolded monomers are incorporated into R15.5 curli. **(b)** Time constants of the single exponential decay functions at the different pH values. R15.5 elongation rates show little pH dependence in the pH 4-8 range. For each pH condition experiment was conducted once (n=1). Data are presented as mean +/- SE. Source data are provided as a Source Data file.

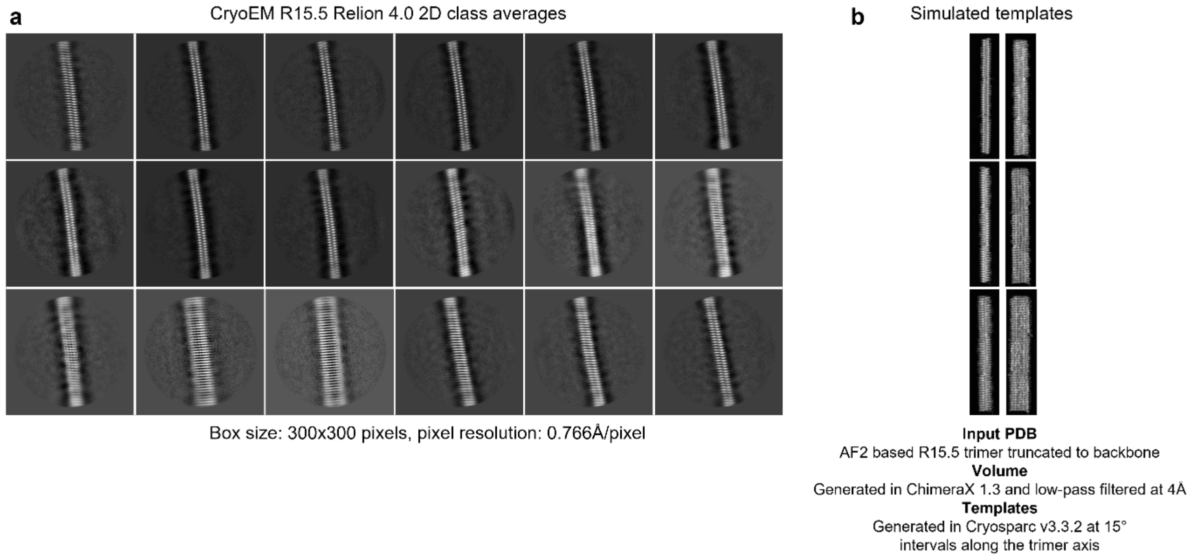

Supplementary Figure 13: CryoEM class averages of R15.5 fibrils. (a) Full set of RELION 4.0 2D class averages of R15.5 fibrils; (b) Simulated templates based on an R15.5 AF2 trimer model, low-pass filtered at 4Å. Projections were generated at 15° degree intervals along the trimer axis.

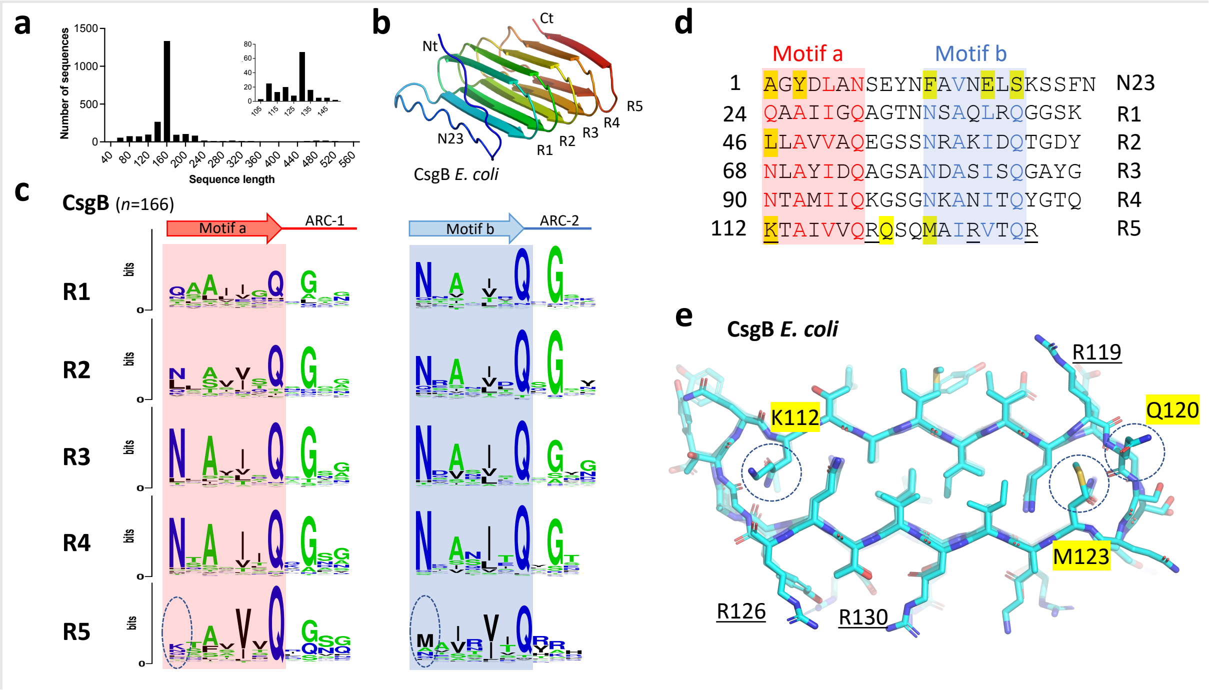

Supplementary Figure 14. Signature motif and structure of minor curli subunits CsgB. (a) Sequence length distribution of RefSeq entries annotated as CsgB (*n*=2296). CsgB sequences show a more narrow length distribution than CsgA, with 95% of sequences falling in the 80 to 240 amino acid interval, corresponding to 4 to 8 curlin repeats. Inset, length distribution of CsgB mature domain sequences encompassing five curli repeats and downscaled to a redundancy of <90% pairwise sequence similarity (*n*=166). **(b)** Alphafold2 model of *E. coli* CsgB as representative CsgB sequence, consisting of a 23 residue secretion signal (N23) and five curlin repeats (R1 to R5), shown as ribbon diagram and colour blue to red from N- to C-terminus (Nt, Ct, resp.) **(c)** Sequence logo of the CsgB sequences, based on multiple sequence alignment of *n*=166 sequences annotated as CsgB, and downscaled to a redundancy of <90% pairwise sequence similarity. To facilitate global alignment and MSA generation, the dataset was restricted to CsgB variants that have five curlin repeats (i.e. lengths between 100 and 160 amino acids to allow for variable lengths of the N23 region at the N-terminus). For clarity, the N-temrinal regions of the mature domain (N22/N23) were removed from the sequence logo due to low similarity or their absence (i.e. 36 out 166 sequences). Sequences logos were generated corresponding to the motif a – arc1 and motif b – arc2 kernels in the R1, R2, R3, R4 and R5 repeats (as indicated). **(d)** Sequence of *E. coli* CsgB mature domain (i.e. lacking its signal peptide) colour coded to highlight motif a and motif b, and with residues defiant of the canonical curlin kernel highlighted in yellow. **(e)** Alphafold2 model of EcCsgB, shown in stick representation viewed along the fiber axis with repeat R5 in front. Highlighted residues (K112, Q120 and M123) deviate from the canonical amyloid kernel and result in a disruption and structural mismatch of the otherwise homotypic interactions in the amyloid core.

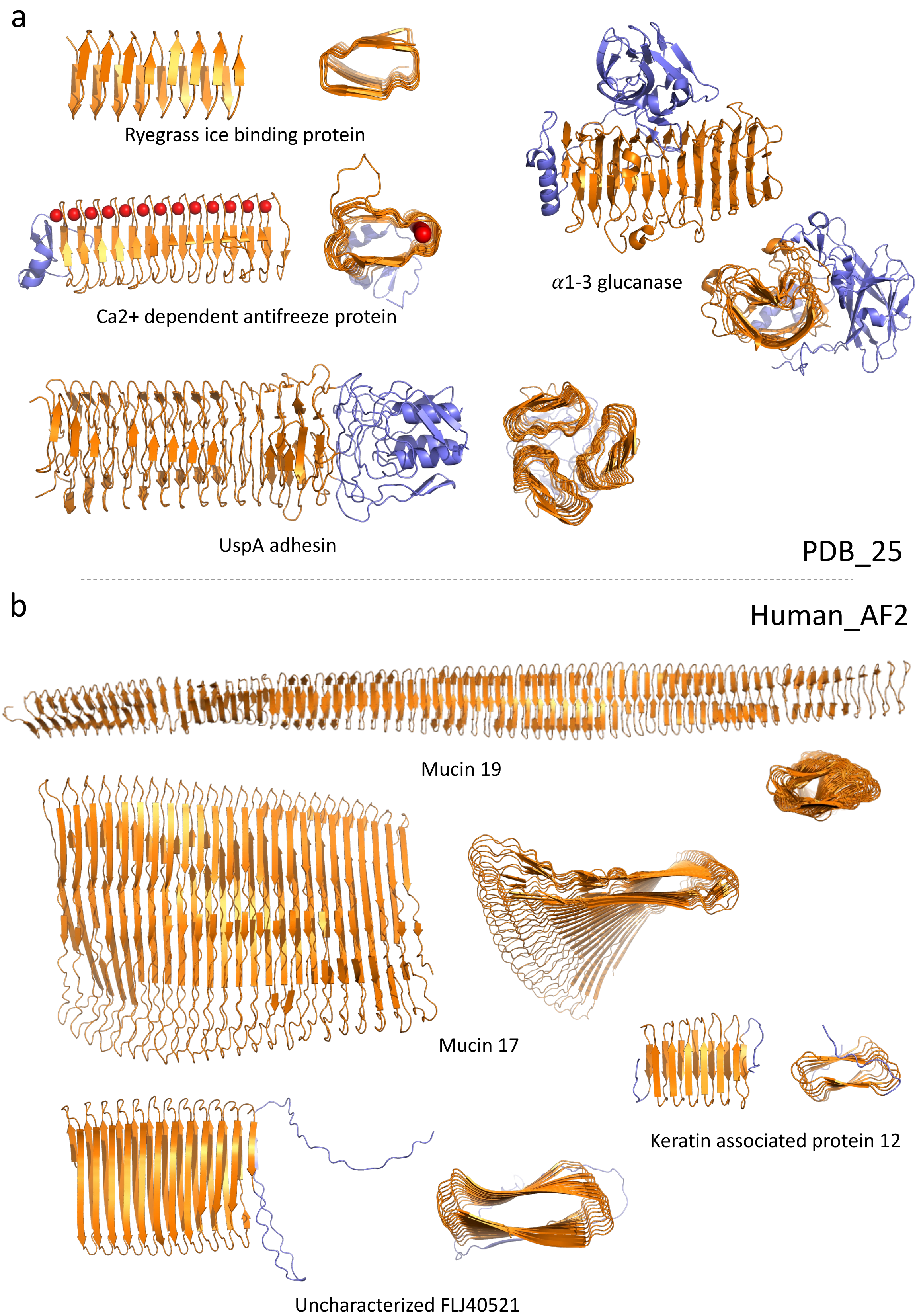

Supplementary Figure 15. Structural comparison of β-solenoid proteins and curli. Represenativeβ-solenoid proteins identified by a structural similarity search of the PDB_25 (**a**) or human Alphafold protein structure database (**b**), using the Dali server (ref) and the CS-class curlin

subunit A0A0E3UX01 (i.e. R15.5). Full results and similarity scores are found in Supplementary Table 2. Structural similarity searches of the full Alphafold protein structure database results in several hundred hits with high Z-score. Supplementary Table 2 shows high similarity hits (Z-score > 6) of the human proteome. Shown representatives the Ryegrass *Lolium perenne* ice-binding protein [http://doi.org/10.2210/pdb3ULT/pdb], the *Marinomonas primoryensis* Ca^{2+}-dependent antifreeze protein ([http://doi.org/10.2210/pdb3P4G/pdb]; Ca^{2+} shown as red spheres), the *Moraxella catarrhalis* adhesin USPA1 [http://doi.org/10.2210/pdb3PR7/pdb] and the *Niallia circulans* α1,3-glucanase [http://doi.org/10.2210/pdb5ZRU/pdb] for the PDB_25 list, and Alphafold models of human mucin-17 (Q685J3), mucin-19 (Q7Z5P9), suprabasin (Q6UWP8), uncharacterized protein FLJ40521 (Q87P7) and keratin associated protein 12-2 (P59991). Models are shown in stick representation, with the solenoid core colored orange. Mostβ-solenoid proteins show a sterically capping domains (blue) or a structural mismatch between the edge and the central arcs of the solenoid core, likely to avoid head-to-tail or tail-to-tail polymerization.

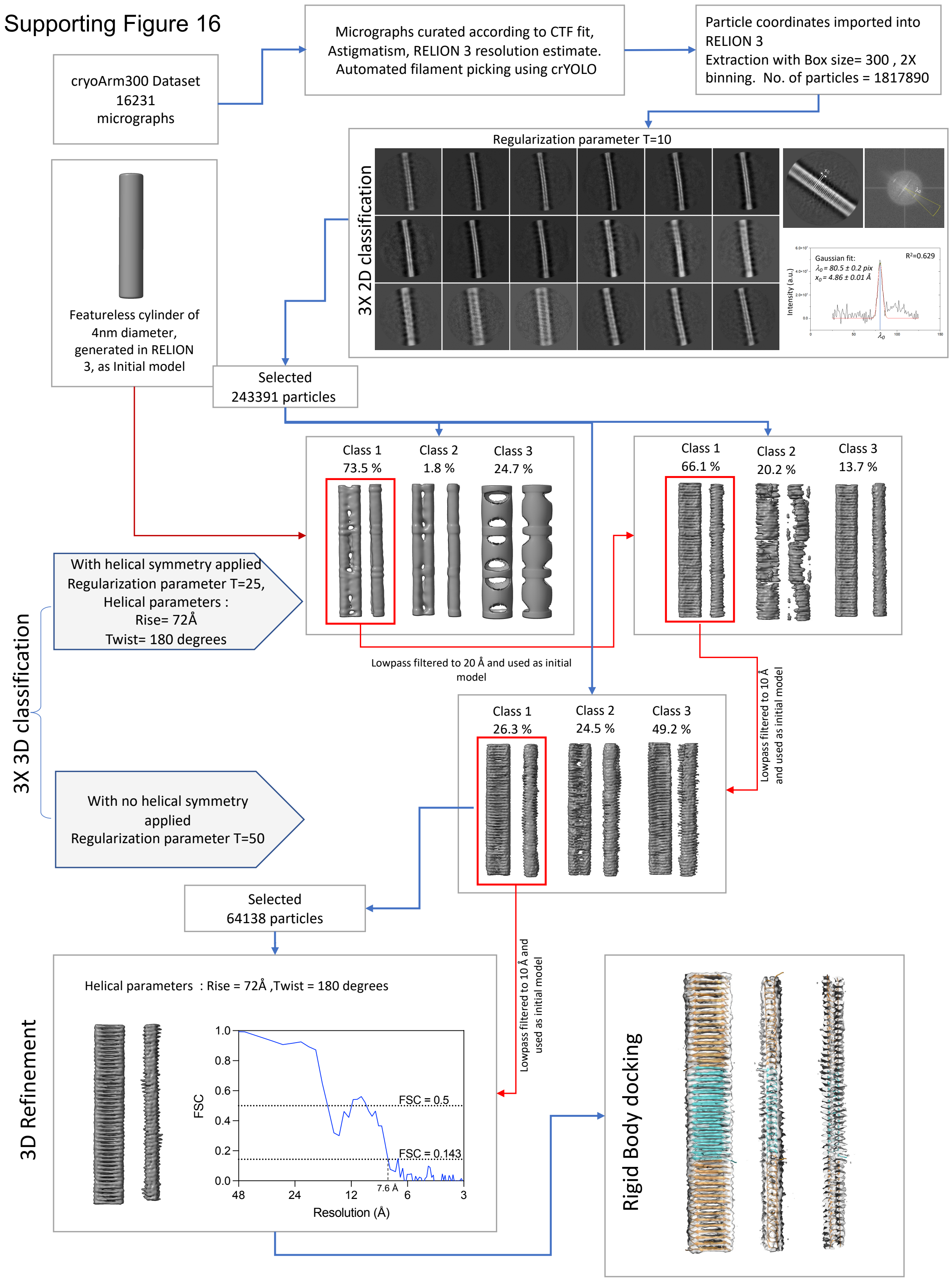

Supplementary Figure 16: EM-processing workflow of recombinant R15.5 protofibrils grown *in vitro*.
